# Somatic mosaicism in the δ-aminolevulinate dehydratase gene causing late-onset porphyria with erythroid-driven pathogenesis

**DOI:** 10.1016/j.ymgmr.2026.101320

**Published:** 2026-05-14

**Authors:** Timofei Vizerov, Nina Demina, Rodion Ponomarev, Elena Lukina, Olesya Pshenichnikova, Daria Selivanova, Vyacheslav Tabakov, Oxana Ryzhkova

**Affiliations:** a Research Centre for Medical Genetics, 1 Moskvorechye Street, Moscow 115478, Russian Federation; b National Medical Research Center for Hematology^,^ Novy Zykovsky Proezd, 4, Moscow 125167, Russian Federation

**Keywords:** δ-Aminolevulinic acid dehydratase deficiency, ALAD porphyria, Acute hepatic porphyria, Somatic mosaicism, Whole-genome sequencing

## Abstract

δ-Aminolevulinate dehydratase deficiency porphyria (ADP) is an extremely rare autosomal recessive disorder, with only 12 previously reported cases. It is characterized by the accumulation of delta-aminolevulinic acid (ALA), which is associated with neurovisceral symptoms.

We report the first case of ADP in Russia with late-onset symptoms and unique genetic findings. A 47-year-old Russian woman experienced recurrent abdominal pain, tachycardia, hypertension, and neuropathy onset at age 38. Biochemical tests revealed a marked and persistent elevation of urinary ALA, whereas PBG levels showed only moderate and episodic increases.

Whole-exome sequencing (WES) revealed two variants in the *ALAD* gene: a novel heterozygous variant c.299dup, p.(Ala101SerfsTer3) and a mosaic (19% of reads) c.415G>A, p.(Gly139Arg). The c.299dup variant was detected in blood and fibroblasts, while the c.415G>A variant was found only in blood, confirming its mosaic state. The c.415G>A variant was recently reported in a neonatal ADP case as a paternally inherited allele, in contrast with its *de novo* mosaic origin in our patient.

Somatic mosaicism has not been previously described for this disease. Accumulating evidence, including the findings from our case, points to a significant contribution of the erythroid lineage to the disease pathogenesis, suggesting that ADP involves both hepatic and erythroid components, whose relative contributions depend on the tissue-specific genotype and other precipitating factors.

## 1 Introduction

δ-Aminolevulinate dehydratase deficiency porphyria (ADP; OMIM 612740), also known as Doss porphyria or plumboporphyria, is the rarest of the eight porphyrias. Our comprehensive literature search identified a total of 12 previously reported patients with a clinical and biochemical diagnosis of ADP [1], [2], [3], [4], [5], [6], [7], [8], [9], [10], [11], [12], [13], [14], [15], [16], [17], [18]. Key biochemical and genetic characteristics of these cases are summarized in Table 1, while a complete detailed comparison is provided in Supplementary Table S1.

**Table 1:** Biochemical and genetic characteristics of reported ADP patients.

Case ID	Ref.	Country	Year of report	Sex/age of onset (yrs)	Erythrocyte ALAD activity (% of normal)	Urinary ALA level (*vs* normal)	Allele 1⁎	Allele 2⁎	Origin of allele 1	Origin of allele 2
ADP1	[1], [2], [3], [4]	Germany	1979	Male/15	< 1%	24×	c.718C>T, p.(Arg240Trp)	c.820G>A, p.(Ala274Thr)	Maternal	n.a.⁎⁎⁎
ADP2	[1], [2], [3], [4]	Germany	1979	Male/15	< 1%	24×	c.457G>A, p.(Val153Met)	c.818_819del, p.(Leu273ArgfsTer22)	Maternal	Paternal
ADP3	[5], [6]	Sweden	1987	Male/<1	< 2%	250×	c.397G>A, p.(Gly133Arg)	c.823G>A, p.(Val275Met)	Maternal	Paternal
ADP4	[7], [8]	Belgium	1989	Male/63	1%	27×	c.177G>C, p.(Lys59Asn) and c.397G>A, p.(Gly133Arg)	WT	Inherited	Inherited
ADP5	[9]	Germany	2004	Male/15	10%	32×	c.114-11C>A, p.?	c.114-11C>T, p.?	Maternal	Paternal
ADP6	[10], [11], [12]	USA	2006	Male/12	< 8%	29×	c.265G>A, p.(Glu89Lys)	c.394 T>C, p.(Cys132Arg)	Paternal	Maternal
ADP7	[13], [14]	Germany	2013	Male/Birth	12%	10×	c.724G>A, p.(Val242Ile)	c.839G>A, p.(Gly280Glu)	Paternal	Maternal
ADP8	[15]	Netherlands	2019	Male/2 days	10%	11×	c.114-11C>T, p.?	c.164 + 2 T>A, p.?	Maternal	Paternal
ADP9	[14], [16]	Italy	2024	Female/5	3%	30×	c.440_441delinsTT, p.(Arg147Leu)	c.440_441delinsTT, p.(Arg147Leu)	Maternal	Paternal
ADP10	[14], [16]	Italy	2024	Male/8	6%	4×	c.440_441delinsTT, p.(Arg147Leu)	c.440_441delinsTT, p.(Arg147Leu)	Maternal	Paternal
ADP11	[17]	France	2024	Male/3	n.a.	15×	c.37C>T, p.(His13Tyr)	c.37C>T, p.(His13Tyr)	Maternal	Paternal
ADP12	[18]	USA	2024	Male/3 days	3.8 nmol/L/s⁎⁎	10×	c.415G>A, p.(Gly139Arg)	c.397G>A, p.(Gly133Arg)	Maternal	Paternal
ADP13	[Present study]	Russia	2024	Female/38	n.a.	11×	c.415G>A, p.(Gly139Arg)	c.299dup, p.(Ala101SerfsTer3)	Somatic	Inherited (not maternal⁎⁎⁎)

Note: This table summarizes the clinical presentation, key biochemical findings, and allelic origin for all known ADP cases (ADP1–ADP13).

Abbreviations: Ref.: reference; n.a.: not applicable/available; ALA: δ-aminolevulinic acid; ALAD: δ-aminolevulinic acid dehydratase; WT: wild type.

⁎ Variants are described using the *ALAD* transcript NM_000031.6.

⁎⁎ Measured post-transfusion; percentage of normal not reported.

⁎⁎⁎ Father's sample not available for segregation analysis.

ADP belongs to the group of acute hepatic porphyrias (AHP), along with acute intermittent porphyria (AIP), variegate porphyria (VP), and hereditary coproporphyria (HCP). Each of these results from deficient activity of a different enzyme in the heme biosynthesis pathway. ADP is an autosomal recessive disorder, whereas AIP, VP, and HCP are inherited in an autosomal dominant pattern with variable penetrance [19].

Heme synthesis occurs in hepatocytes and erythroid precursor cells. δ-aminolevulinic acid dehydratase (ALAD) is the second enzyme in the heme synthesis pathway, following δ-aminolevulinic acid synthase (ALAS). Two isoforms of ALAS exist in the body: one is expressed in erythroid precursor cells (ALAS2), while the other (ALAS1) is expressed predominantly in liver cells. About 20% of heme synthesis in the body occurs in the liver, and is controlled through feedback regulation of ALAS1. Most of the remaining 80% of heme production occurs in the bone marrow. The activity of ALAS2 in the bone marrow, unlike ALAS1 in the liver, is regulated by iron and erythropoietin [20]. ALAD catalyzes the conversion of δ-aminolevulinic acid (ALA) to porphobilinogen (PBG).

In normal cells, ALAD activity is in excess, so partial loss of its activity is not accompanied by clinical symptoms [21]. Severe ALAD deficiency in ADP or its inhibition in other conditions such as lead poisoning [22] and hereditary tyrosinemia 1 [23], [24] leads to the accumulation of ALA and coproporphyrin III in plasma and urine, and zinc protoporphyrin in erythroid cells, with little or no elevation in PBG, and similar neuropathic symptoms [25].

ADP attacks are accompanied by abdominal pain, motor neuropathy, and other neurological, psychiatric, and cardiovascular symptoms. Patients with early-onset disease may present with psychomotor delay, sensorineural hearing loss, resistant arterial hypertension, and respiratory failure. All previously described patients, regardless of the age of onset, developed sensorimotor neuropathy (Table 1).

The detection of *ALAD* variants classified as pathogenic or likely pathogenic according to the American College of Medical Genetics and Genomics (ACMG) criteria [26] is crucial for the diagnosis of ADP [27].

Currently, the accepted treatment for ADP is the same as for other forms of AHP. When administered intravenously, hemin rapidly suppresses the expression of hepatic ALAS1, thereby slowing the synthesis and accumulation of ALA and PBG [19], with individual case reports providing clinical evidence for its efficacy in ADP. While the use of intravenous and oral glucose solutions is conventionally recommended for managing mild attacks, based on the biochemical rationale that glucose can indirectly prevent the translation of ALAS1 [20], clinical evidence for its efficacy specifically in ADP is lacking. However, due to its rarity, experience supporting the effectiveness of all treatments for ADP is limited.

## 2 Material and methods

### 2.1 Clinical data

The proband was examined at the National Medical Research Center for Hematology of the Ministry of Health of the Russian Federation and the Research Centre for Medical Genetics. Her medical records, including clinical history and the results of instrumental and laboratory tests, were reviewed. The study was approved by the Ethics Committee of the Research Centre for Medical Genetics, Moscow, Russia (Approval No. 4/1, dated 19 April 2021). All research was conducted in accordance with both the Declarations of Helsinki and Istanbul. Written informed consent was obtained from the patient.

### 2.2 Genetic testing

*ALAD* variants in the proband were identified by whole-exome sequencing (WES) analysis. DNA extraction and WES were performed at the Laboratory of Molecular Genetic Diagnostics №3 of the Research Centre for Medical Genetics using the facilities of the "Genome" Shared Resource Centre. A fibroblast culture was grown at the "Biobank" Core Facility of the Research Centre for Medical Genetics. The WGS analysis was a collaborative effort with Biotech Campus LLC under the national genetic initiative "100,000 + Я".

DNA was extracted using the QIAamp DNA Blood Mini Kit (Qiagen, Germany). WES was performed on a DNBSEQ-400 next-generation sequencer (MGI, China) using a paired-end 2 × 150 bp sequencing. Sample preparation was performed using the Nanodigmbio NadPrep DNA Library Kit (for MGI) and the Nanodigmbio NEXome Plus Panel v1.0 (Nanodigmbio, China).

Whole-genome sequencing (WGS) was performed on a DNBSEQ-T7 genetic analyzer using a PE150. A PCR-free protocol with enzymatic fragmentation (MGI) was used for library preparation.

Nanopore sequencing was performed on an Oxford Nanopore PromethION instrument (Oxford Nanopore technology, Oxford, UK) equipped with R10.4.1 (Kit 14, Q20+) flow cells.

For WES of peripheral blood DNA, the mean coverage was 84×, with 1.79% of target fragments having coverage below 10×. For WES of skin fibroblast DNA, the mean coverage was 302×, with 1.7% of target fragments having coverage below 10×. For WGS of peripheral blood DNA, the mean coverage was 56×.

The identified variants were named according to the HGVS nomenclature guidelines, version 21.1.3 [28]. Automated bioinformatic analysis of high-throughput sequencing data was performed using the custom in-house pipeline NGS-data-Omics (state registration no. 2025685783, Research Centre for Medical Genetics) [29].

The clinical significance of the identified variants was evaluated based on the Russian guidelines for the interpretation of data obtained by massive parallel sequencing (MPS) [30], the joint consensus recommendation of the ACMG and the Association for Molecular Pathology [26] and ClinGen Variant Classification Guidance [31].

To verify and confirm the presence of variants and for segregation analysis, the Sanger sequencing method was used with forward and reverse primers. Peripheral blood samples from the proband, her mother, sister, and daughter were available. Sequencing was performed according to the manufacturer's protocol on an ABI Prism 3100 instrument (Applied Biosystems/Thermo Fisher Scientific, Waltham, MA, USA). Custom primers for *ALAD* (NM_000031.6) were used: ALAD_F: GAGAAGGCCAGGGCTATGCT and ALAD_R: CAGAGGGTGGCCTTCAGAACTC.

## 3 Results

### 3.1 Clinical presentation and initial investigations

The proband is a 47-year-old female with normal growth and development who gave birth to a healthy daughter at age 23. The first episode of abdominal pain occurred at the age of 38, 10 days after a planned laparoscopic myomectomy performed for a submucosal uterine myoma. Over the subsequent 8 years, the patient experienced recurrent attacks of abdominal pain, accompanied by tachycardia, arterial hypertension, nausea, and constipation. The attacks gradually increased in frequency, from once to five times a year. During this time, she had several hospitalizations and a diagnostic laparoscopy for a suspected acute abdomen.

**Fig. 1:**
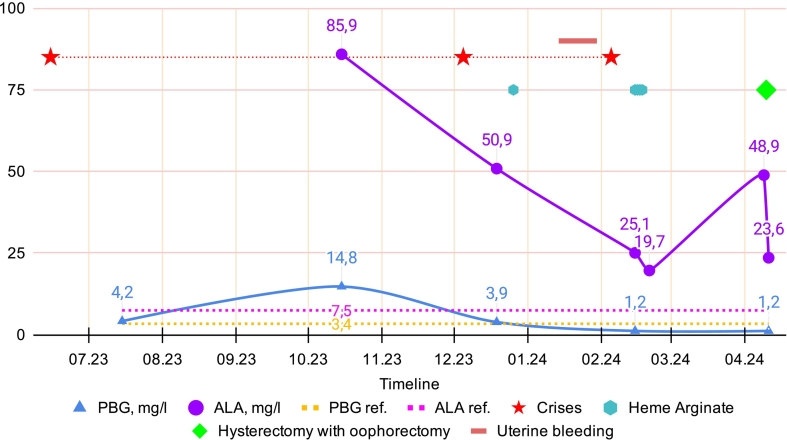
Biochemical and clinical profile. Urinary concentrations of ALA (purple circles and line) and PBG (blue triangles and line) over time, with reference ranges indicated by dashed lines (pink: ALA; orange: PBG). Red stars denote occurrence of acute porphyria attacks; blue hexagons indicate daily heme arginate administrations (one dose in Dec 2023 and a 4-day course in Feb 2024); red horizontal bar indicates the uterine bleeding episode; green diamond marks hysterectomy with oophorectomy.

At the age of 46, she developed muscle weakness and was examined at the Research Center of Neurology in Moscow, where a diagnosis of acute porphyria was first suspected. The patient was hospitalized several times in the Department of Hematology and Chemotherapy for Orphan Diseases at the National Medical Research Center for Hematology in Moscow for examination and treatment. Multiple tests revealed periodic slight increases in PBG concentration and much more marked increases in ALA concentration in her urine (Fig. 1). During acute attacks, she was treated with intravenous heme arginate: a single 125 mg dose was administered in December 2023, followed by a four-day course (125 mg daily) in February 2024, which resulted in clinical improvement. Notably, this February 2024 attack was preceded by a prolonged episode of intensive uterine bleeding occurring from January 14 to 30. The absolute concentration of urinary ALA did not strictly correlate with occurrence of acute attacks; for instance, the patient remained asymptomatic in October 2023 despite a peak ALA level of 85.9 mg/L. However, her symptomatic episodes showed a clear response to heme arginate therapy, with symptom resolution coinciding with a significant reduction in ALA levels (Fig. 1). Blood lead levels were not elevated.

Neurological examination revealed signs of sensorimotor polyneuropathy: peripheral tetraparesis, swelling of the hands, absence of periosteal reflexes in the left arm, absence of Achilles reflexes, and inability to walk on her heels and toes. The patient had chronic normocytic, normochromic anemia (hemoglobin 92–118 g/L), which was exacerbated by uterine bleeding associated with a recurrent uterine myoma. Notably, iron studies revealed no deficiency, confirming the anemia was not iron-deplete: ferritin levels ranged from 268.1 to 312.3 ng/mL, and serum iron levels were normal (14.56–30.32 μmol/L). According to available medical records, there was no documentation of blood transfusions or specific anemia treatments during the observed period. Her gynecological history was also notable for secondary amenorrhea, for which she had been receiving continuous hormone replacement therapy (Femoston) since 2019. Furthermore, laboratory tests revealed stage 3a chronic kidney disease, with baseline creatinine levels of 104.5–110.5 μmol/L (reference range 49.0–90.0) that transiently peaked at 130.2 μmol/L during the acute attack in February 2024. C-reactive protein was also elevated at 10.44 mg/L (reference range 0.0–5.0).

Molecular genetic testing of the *PPOX* (VP), *CPOX* (HCP), and *ALAD* (ADP) genes was performed by Sanger sequencing. Sanger sequencing of the *ALAD* gene revealed a heterozygous variant (NM_000031.6: c.299dup, p.(Ala101SerfsTer3)). WES was recommended following a consultation at the Research Centre for Medical Genetics.

In April 2024, the patient underwent a planned hysterectomy with oophorectomy for uterine myoma and associated uterine bleeding. During this hospitalization, her urinary ALA concentration remained elevated preoperatively, while the PBG concentration was within the reference range. Urinary ALA levels showed a marked decline following this surgery (Fig. 1). She had no active complaints related to ADP during this hospitalization.

Over the subsequent 12 months, the patient experienced more than 10 acute porphyria attacks without any identifiable provoking factors. Baseline urinary ALA levels remained persistently elevated, exceeding 100 mg/L during exacerbations. The patient was transitioned to prophylactic heme arginate infusions (3 mg/kg/day for two consecutive days every three weeks), which provided only short-term relief (approximately one week) and failed to prevent recurrent attacks. Consequently, her medical team is considering ALAS1-targeted RNA interference therapy (givosiran) as the next therapeutic step.

### 3.2 Genetic analyses

Upon referral to the Research Centre for Medical Genetics, the proband underwent WES on DNA extracted from whole blood to search for pathogenic variants associated with porphyria, as well as with other hereditary diseases with similar phenotypic manifestations.

**Fig. 2:**
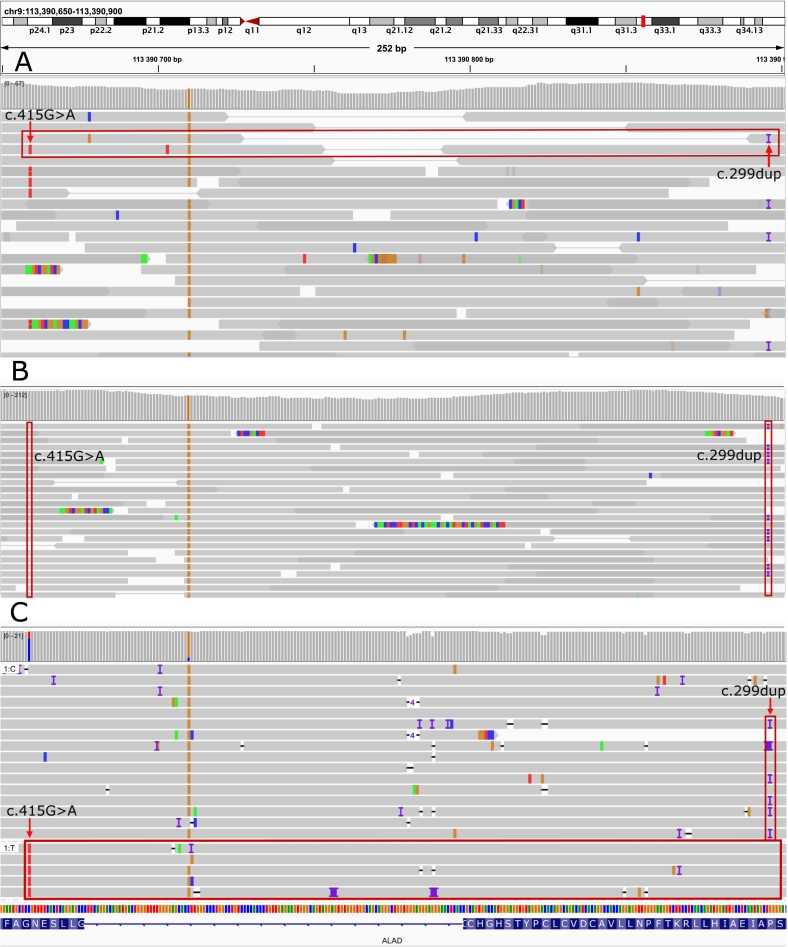
Genetic characterization of compound heterozygous *ALAD* variants and somatic mosaicism. Screenshots from the Integrative Genomics Viewer (IGV) showing: (A) WES of peripheral blood DNA reveals two pathogenic *ALAD* variants: c.299dup and c.415G>A in trans configuration, supported by a spanning read pair. (B) WES of skin fibroblast-derived DNA shows the presence of c.299dup but absence of the c.415G>A variant, confirming its somatic mosaic origin. (C) Long-read WGS of blood DNA validates the trans configuration of the two variants.

Two variants were found in the *ALAD* gene: the pathogenic variant NM_000031.6: c.415G > A, p.(Gly139Arg), recently described in another patient with ADP [18], in a suspected mosaic state (19% of reads), and a novel pathogenic variant NM_000031.6: c.299dup, p.(Ala101SerfsTer3) in a heterozygous state. The detailed justification of the ACMG classification criteria for these variants is provided in Supplementary Material 1. Although the variants were located further apart than the average read length (236 bp), a single read pair suggested they were in a trans-configuration (Fig. 2, A).

**Fig. 3:**
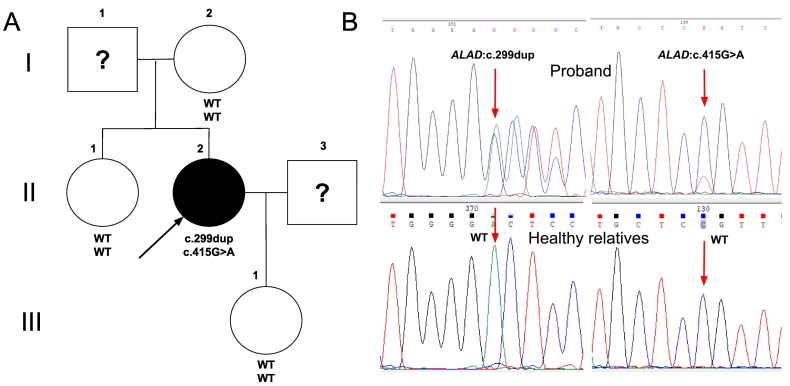
Segregation analysis of *ALAD* variants in the proband and family members. (A) Pedigree showing the proband (black circle, II-2) with compound heterozygous *ALAD* variants. The proband's mother (I-2), sister (II-1), and daughter (III-1) are wild-type for both alleles. Father (I-1) and husband (II-3), marked with a question mark (?), were not available for genetic testing. (B) Sanger sequencing chromatograms at positions c.299 (left) and c.415 (right). The proband shows a heterozygous frameshift variant c.299dup and a mosaic missense variant c.415G>A. Healthy relatives show only wild-type alleles at both positions.

To confirm the compound heterozygous state of the variants and to determine their inheritance pattern, a segregation analysis was performed. Biological material was available from the proband, her mother, her sister, and her daughter. Neither variant was detected in the available family members, including one parent (Fig. 3), suggesting that at least one of the variants arose *de novo* in the proband.

We performed WES with increased coverage on DNA extracted from the proband's skin fibroblasts. The c.299dup variant was detected in both blood and skin fibroblasts, confirming its germline origin. In contrast, the c.415G>A variant was absent in fibroblasts, confirming our hypothesis of somatic mosaicism for this variant (Fig. 2, B). Given this evidence, we infer that the c.299dup variant was most likely inherited from the father, while the c.415G>A variant arose *de novo* as a somatic mosaic mutation.

To exclude the presence of other variants in the introns of the *ALAD* gene that could be a possible cause of the disease, short-read and long-read WGS was performed on DNA extracted from the proband's whole blood. The WGS results excluded the presence of potentially pathogenic intronic variants and also confirmed the trans-configuration of the variants identified by WES (Fig. 2, C).

Comprehensive information on the identified and previously reported *ALAD* causative variants in ADP, including population frequencies, is provided in Table 2.

**Table 2:** Genomic, population, and computational profiling of *ALAD* variants identified in reported ADP cases.

Exon	HGVSc	HGVSp	Case ID	gnomAD v3.1.2 AF (AC) [32]	gnomAD v4.1.0 AF (AC) [32]	GDB AF (AC) [33]	REVEL [34]	splice_ai [35]	ACMG/AMP classification [[26], [31]]
2/12	c.37C>T	p.(His13Tyr)	ADP11 [17]	6.6 × 10^−6^ (1)	1.2 × 10^−6^ (2)	n.a.	0.86	n.a.	Likely Pathogenic (PM2, PP3, PM3, PP4)
2/11	c.114-11C>A	p.?	ADP5 [9]	n.a.	1.2 × 10^−6^ (2)	n.a.	n.a.	0.84	Pathogenic (PVS1, PM2, PM3, PP4)
2/11	c.114-11C>T	p.?	ADP5 [9] ADP8 [15]	n.a.	3.1 × 10^−6^ (5)	4.1 × 10^−6^ (1)	n.a.	n.a.	Likely Pathogenic (PM2, PM3, PP4)
3/11	c.164 + 2 T>A	p.?	ADP8 [15]	n.a.	7.4 × 10^−6^ (12)	n.a.	n.a.	0.99	Pathogenic (PVS1, PM2, PM3, PP4)
5/12	c.265G>A	p.(Glu89Lys)	ADP6 [10], [11], [12]	n.a.	2.5 × 10^−6^ (4)	n.a.	0.52	n.a.	Likely Pathogenic (PM2, PP3, PM3, PP4)
5/12	c.299dup	p.(Ala101SerfsTer3)	ADP13 [Present study]	n.a.	n.a.	n.a.	n.a.	n.a.	Pathogenic (PVS1, PM2, PP4)
5/12	c.394 T>C	p.(Cys132Arg)	ADP6 [10], [11], [12]	n.a.	n.a.	n.a.	0.94	n.a.	Likely Pathogenic (PM2, PP3, PM3, PP4)
5/12	c.397G>A	p.(Gly133Arg)	ADP3 [5], [6] ADP4 [7], [8] ADP12 [18]	4.6 × 10^−5^ (7)	1.6 × 10^−5^ (25)	4.1 × 10^−6^ (1)	0.84	n.a.	Pathogenic (PM2, PM3, PP3, PP4)
6/12	c.415G>A	p.(Gly139Arg)	ADP12 [18] ADP13 [Present study]	n.a.	1.2 × 10^−6^ (2)	8.3 × 10^−6^ (2)	0.77	n.a.	Pathogenic (PM2, PM3, PM6, PP3, PP4)
6/12	c.440_441delinsTT	p.(Arg147Leu)	ADP9 [14], [16] ADP10 [14], [16]	n.a.	n.a.	n.a.	n.a.	n.a.	Likely Pathogenic (PM2, PP3, PM3, PP4)
6/12	c.457G>A	p.(Val153Met)	ADP2 [1], [2], [3], [4]	n.a.	n.a.	n.a.	0.72	n.a.	Likely Pathogenic (PM2, PP3, PM3, PP4)
10/12	c.718C>T	p.(Arg240Trp)	ADP1 [1], [2], [3], [4]	1.3 × 10^−5^ (2)	1.1 × 10^−5^ (17)	4.1 × 10^−6^ (1)	0.71	n.a.	Likely Pathogenic (PM2, PM3, PP3, PP4)
10/12	c.724G>A	p.(Val242Ile)	ADP7 [13], [14]	1.5 × 10^−5^ (23)	1.8 × 10^−4^ (286)	2.6 × 10^−4^ (63)	0.24	n.a.	Likely Pathogenic (PS3, PM2, PM3, PP4)
11/12	c.818_819del	p.(Leu273ArgfsTer22)	ADP2 [1], [2], [3], [4]	n.a.	n.a.	n.a.	n.a.	n.a.	Pathogenic (PVS1, PM2, PM3)
11/12	c.820G>A	p.(Ala274Thr)	ADP1 [1], [2], [3], [4]	6.6 × 10^−6^ (1)	6.2 × 10^−6^ (10)	8.3 × 10^−6^ (2)	0.81	n.a.	Likely Pathogenic (PM2, PP3, PM3, PP4)
11/12	c.823G>A	p.(Val275Met)	ADP3 [5], [6]	1.3 × 10^−5^ (1)	1.7 × 10^−5^ (27)	2.5 × 10^−5^ (6)	0.83	n.a.	Likely Pathogenic (PM2, PM3, PP3, PP4)
11/12	c.839G>A	p.(Gly280Glu)	ADP7 [13], [14]	n.a.	n.a.	n.a.	0.94	n.a.	Likely Pathogenic (PM2, PP3, PM3, PP4)

Note: This table summarizes the genomic location, predicted protein consequences (referenced to the *ALAD* transcript NM_000031.6), and population frequencies of all reported variants. The data are ordered sequentially by their location across the *ALAD* exons (5′ to 3′ direction). Computational pathogenicity scores and standardized ACMG/AMP classifications are provided to support the clinical interpretation of each variant.

Abbreviations: HGVSc/HGVSp: Human Genome Variation Society nomenclature for coding DNA (c.) and protein (p.) sequences; gnomAD: Genome Aggregation Database (v3.1.2 and v4.1.0); AF: allele frequency; AC: allele count; GDB: Database of population frequencies of genetic variants of the population of the Russian Federation; REVEL: Rare Exome Variant Ensemble Learner (pathogenicity score for missense variants; scores >0.75 indicate likely pathogenic); SpliceAI: artificial intelligence tool for predicting splice site alterations (scores >0.8 indicate a high probability of impact on splicing); ACMG/AMP: American College of Medical Genetics and Genomics/Association for Molecular Pathology clinical variant classification. Alphanumeric codes in parentheses (*e.g.*, PVS1, PM2) represent specific lines of evidence used for classification.

## 4 Discussion

Most previously described patients with ADP had an early onset of the disease, from birth to 16 years of age. We have collected the most up-to-date and comprehensive information available on all reported ADP cases. All but two were found to have variants in the *ALAD* gene on each allele, inherited from their parents (Table 1).

The Belgian patient (ADP4) [7], [8] is particularly noteworthy. Two variants in the *ALAD* gene were found on the same allele (in cis). One of them is a benign polymorphism. In this patient, ALAD activity was approximately 1% in erythrocytes but 20% in lymphoblastoid cells. His relatives who were carriers of these variants had ALAD activity of about 50%. His symptoms developed at the age of 63 after he was diagnosed with polycythemia vera. The authors hypothesized that the clinical picture of ADP was caused by the expansion of the polycythemic hematopoietic clone that carried the mutant allele [8]. In this case, several mechanisms could potentially explain the observed tissue-specific ALAD deficiency. As suggested by Lahiji et al. [11], acquired cytogenetic abnormalities common in polycythemia vera, such as trisomy 9 or other rearrangements of chromosome 9, could have further increased the ALAD deficiency in the hematopoietic lineage. Alternatively, it remains possible that a second pathogenic variant was present but undetected due to the limitations of earlier analytical methods.

The ADP13 patient we describe (ADP13) is notable for the late onset of attacks, as well as for the presence of a somatic mosaic variant. Dermal cells, from which fibroblasts originate, and hematopoietic cells both develop from the mesoderm, whereas liver cells develop from the endoderm. Knowing that the NM_000031.6: c.415G>A, p.(Gly139Arg) variant is absent in fibroblasts but present in blood cells, we can conclude that it arose after the differentiation of the mesoderm into somites and splanchnotome [36]. This implies that endodermal cells, including hepatocytes, should only have one mutant allele with the NM_000031.6: c.299dup, p.(Ala101SerfsTer3) variant. We cannot yet confirm this with a molecular genetic study, as it would require a liver biopsy, which is not clinically indicated in this patient.

Since the somatic variant is detected at 19% VAF in bulk peripheral blood DNA, which is extracted from nucleated leukocytes, the mutation must be present in the leukocyte lineage and is not restricted solely to erythropoietic cells. Therefore, the mutation likely originated in a multipotent hematopoietic stem cell (HSC) or a common myeloid progenitor. While we cannot definitively exclude the possibility that the mutation arose even earlier during embryogenesis without biopsies of other mesenchyme-derived tissues like heart or smooth muscle, the late clinical onset strongly favors a localized hematopoietic origin. We hypothesize that a single HSC acquired the somatic variant and underwent gradual clonal expansion over decades. Once this mutant clone expanded to account for approximately 38% of the hematopoietic output, reflecting the 19% VAF, the total ALAD enzymatic capacity in the heavily heme-producing erythroid lineage became limiting for metabolism of ALA in amounts needed for heme synthesis.

Gradual clonal expansion of the somatic variant likely explains the delay in developing symptoms until the fourth decade of life. The underlying somatic mosaicism created a susceptible genetic background, but a specific environmental and physiological triggering factor was required to precipitate the disease onset. In this case, the first acute attack closely followed a laparoscopic myomectomy. Although the exact indication for this initial 2015 surgery and the volume of intraoperative blood loss are undocumented, submucosal myomas frequently manifest with chronic bleeding, which likely established a baseline of continuous compensatory erythropoiesis. While surgical stress and anesthesia classically trigger acute attacks in other AHPs by inducing hepatic ALAS1, the unique genetic landscape of our patient suggests a dual, but predominantly erythropoietic, mechanism. Since the liver harbors only a heterozygous mutation while the bone marrow contains the biallelic defect, it is highly likely that prolonged preoperative uterine bleeding, which presumably necessitated the surgery, stimulated a compensatory wave of erythropoiesis. We hypothesize that acute blood loss during the surgical procedure itself compounded this erythropoietic drive. Concurrently, surgical stress likely induced hepatic ALAS1, transiently overwhelming the single functioning *ALAD* allele in the liver. This combined metabolic overload unmasked the previously subclinical enzyme deficiency. Following this initial event, the patient's recurrent attacks over the subsequent eight years were likely maintained by chronic uterine bleeding from the recurrent uterine myoma. Her recent 2024 records provide direct clinical evidence for this mechanism: a documented episode of prolonged uterine bleeding directly preceded her severe acute attack. Without blood transfusions to suppress marrow activity, and with sufficient iron stores (ferritin up to 312 ng/mL) preventing iron-deficiency limitations, this ongoing bleeding and resulting chronic anemia perpetuated a state of continuous compensatory erythropoiesis, providing a persistent physiological trigger for erythroid ALA overproduction. Therefore, while ADP is classified as an acute hepatic porphyria, the disease in this specific mosaic patient appears to be predominantly driven by the erythroid compartment.

The molecular complexity of *ALAD* variants has been further elucidated by recent *in vivo* functional studies. For instance, Di Pierro et al. [14] dissected the complex allele c.440_441delinsTT found in the Italian patients (ADP9 and ADP10), experimentally confirming that the protein dysfunction is exclusively driven by the missense substitution c.440G>T leading to p.(Arg147Leu), while the adjacent synonymous change does not affect the enzyme. In the same study, the *in vivo* expression of the c.724G>A p.(Val242Ile) variant demonstrated substantial residual enzymatic activity, characterizing it as a hypomorphic allele. This functional observation aligns well with our population database analysis (Table 2), which identifies p.(Val242Ile) as the most frequent causative *ALAD* allele both in the global Genome Aggregation Database [32] with an allele frequency (AF) of 1.8 × 10^−4^ and in the regional Russian Database of population frequencies [33] with an AF of 2.6 × 10^−4^. The relatively higher prevalence of this variant in the general population is likely attributable to its mild functional impact. Consequently, p.(Val242Ile) may represent a more common genetic contributor to ADP when co-inherited with a severe allele, further highlighting the complex allelic interactions underlying the disease.

Targeted therapy with intravenous hemin remains the cornerstone of treatment for many ADP patients. As highlighted by Lahiji et al. [11], hemin was initially reported to be highly successful in several cases, including the first USA case (ADP6), providing significant clinical benefit by dramatically lowering plasma and urinary ALA levels and preventing acute attacks during long-term prophylactic treatment. However, beyond the evidence from ADP4 [7], [8], the major role of the bone marrow in ADP pathogenesis is further supported by specific treatment outcomes. In the Dutch case (ADP8), the authors noted a reduced response of ADP patients to standard therapy for acute hepatic porphyrias with heme preparations. Their patient responded well to therapy aimed at reducing erythroid heme synthesis, which included blood transfusions and hydroxyurea [15]. In a recently described French case (ADP11) [17] and in the investigations by Di Pierro et al. [14], metabolic studies provided additional insights into the response to heme arginate: while hemin reduced urinary ALA, it did not appear to significantly impact ALA concentrations in erythrocytes or cerebrospinal fluid. While it remains to be determined whether the clearance of ALA from these specific compartments is essential for achieving clinical benefit, these biochemical observations suggest that exclusively hepatic-directed therapies may not fully address the systemic accumulation of ALA in cases where the erythroid lineage is a significant contributor.

This clinical perspective is consistent with the recent course of our mosaic proband (ADP13): over the past year, she suffered more than 10 recurrent attacks. In an effort to control the disease, she was recently started on prophylactic heme arginate infusions every three weeks. However, while these infusions provide short-term clinical relief (lasting approximately one week), they ultimately fail to prevent the recurrence of attacks. A strikingly similar clinical pattern was recently reported by Graff et al. in the long-term follow-up of ADP6 [12]. Prophylactic hemin provided only short-term decreases in ALA levels, and givosiran (a drug that specifically suppresses hepatic ALAS1) was completely ineffective in altering the disease course. Clinical evidence for glucose in ADP remains limited [4]. Ultimately, while hemin effectively controls acute attacks in some patients, these varied responses underscore the dual nature of the disease.

ADP is traditionally classified as an acute hepatic porphyria. The hepatic contribution to the disease was recently validated at the molecular level by Lahiji et al. [11], who provided the first direct evidence of hepatic ALAS1 induction by documenting markedly elevated circulating ALAS1 mRNA in an ADP patient. However, a recent comprehensive review noted that "ADP is not readily classified as hepatic or erythropoietic" [37], reflecting the complexity of its pathophysiology.

Accumulating evidence suggests that while ADP is classified as an acute hepatic porphyria, it also has a significant erythroid component. This is supported by the late-onset case described here (ADP13) and patient ADP4, whose symptoms emerged only after polycythemia vera-driven erythroid clonal expansion [8]. Furthermore, multiple reported cases demonstrate a lack of erythrocyte ALA clearance [14], [17] and limited efficacy of hepatic-directed therapies [12], [14], [17]. The prominence of this erythroid component likely varies among patients, being particularly critical in cases with severe mutations or, as in our proband (ADP13), where the biallelic defect is restricted to the bone marrow. Our identification of somatic mosaicism putatively restricted to the hematopoietic lineage provides strong molecular evidence that erythroid-specific ALAD deficiency is sufficient to drive clinical disease. Specifically, it demonstrates that a biallelic defect, composed of one germline and one somatic mutation within the bone marrow, can precipitate the disorder, even in the absence of inherited biallelic germline mutations.

## 5 Conclusions

We report the first case of δ-aminolevulinate dehydratase porphyria in Russia and the first description of somatic mosaicism in this disorder. The patient carries a germline frameshift variant (c.299dup) and a presumably blood-restricted mosaic missense variant (c.415G>A), confirming compound heterozygosity in the hematopoietic lineage. This genetic configuration explains the late disease onset and supports the emerging view that while ADP is an acute hepatic porphyria, its pathophysiology includes a clinically significant erythroid component that varies among cases. Consequently, therapeutic strategies targeting heme synthesis in the bone marrow may be more effective than conventional liver-directed approaches in patients where this erythroid component is prominent, particularly in cases where severe, biallelic ALAD deficiency is restricted to the bone marrow.

The following are the supplementary data related to this article.

Supplementary material 1 Supplementary Table S1. Clinical and biochemical characteristics of reported ADP patients

Supplementary material 2 Supplementary Material 1. Detailed ACMG/AMP pathogenicity classification of identified *ALAD* variants

## CRediT authorship contribution statement

**Timofei Vizerov:** Writing – review & editing, Writing – original draft, Visualization, Methodology, Investigation, Formal analysis, Data curation, Conceptualization. **Nina Demina:** Resources, Investigation. **Rodion Ponomarev:** Resources, Investigation. **Elena Lukina:** Resources. **Olesya Pshenichnikova:** Resources, Investigation. **Daria Selivanova:** Investigation. **Vyacheslav Tabakov:** Resources, Investigation. **Oxana Ryzhkova:** Writing – review & editing, Supervision, Funding acquisition, Conceptualization.

## Informed consent

Written informed consent was obtained from the patient for the publication of this case report and any accompanying tables, figures, or data. The patient explicitly consented to the use of de-identified clinical, biochemical, and genetic information in scientific publications, including international peer-reviewed journals, and acknowledged that the article may be freely accessible on the internet and viewed by the general public. A copy of the signed consent form is available upon request from the corresponding author.

## Ethical approval

The study was conducted in accordance with the principles of the Declaration of Helsinki and was approved by the Ethics Committee of the Research Centre for Medical Genetics (Approval No. 4/1, dated 19 April 2021).

## Funding

The research was carried out within the state assignment of Ministry of Science and Higher Education of the Russian Federation for Research Centre for Medical Genetics.

## Declaration of competing interest

The authors declare that they have no known competing financial interests or personal relationships that could have appeared to influence the work reported in this paper.

## Data Availability

The data that support the findings of this study are not openly available due to patient privacy but are available from the corresponding author upon reasonable request. Any such request would be subject to review by an ethics committee.
