# Supplementary material for: Somatic mosaicism in the δ-aminolevulinate dehydratase gene causing late-onset porphyria with erythroid-driven pathogenesis
Source: Mol Genet Metab Rep. 2026 May 14;47:101320. doi: 10.1016/j.ymgmr.2026.101320 (PMC13181288; doi:10.1016/j.ymgmr.2026.101320)
Supplement: Supplementary material 1 — Supplementary Table S1. Clinical and biochemical characteristics of reported ADP patients [file mmc1.docx]

**Gene Information:**

Gene Symbol: ALAD

RefSeq Transcript: NM_000031.6

Genomic Assembly: GRCh38/hg38

**Variant 1 (Somatic Mosaic)**

HGVS Nucleotide: c.415G>A

HGVS Protein: p.(Gly139Arg)

Genomic Coordinates (hg38): chr9:113390659C>T

Variant Type: Missense

Zygosity in Proband (Blood): Mosaic (approximately 19% of reads)

Zygosity in Proband (Fibroblasts): Absent (Wild-type)

Method of Detection: Whole Exome Sequencing (WES), Whole Genome Sequencing (WGS), confirmed by Sanger sequencing.

Inheritance: Somatic de novo. The variant was not detected in the proband's mother, sister, or daughter. Paternal DNA was not available.

**ACMG/AMP Classification: Pathogenic**

**Applied Criteria:** PM2_Supporting + PM3_Strong + PM6 + PP3 + PP4

**Justification of Applied ACMG/AMP Criteria:**

- **PM2_Supporting:** Variant is absent from, or at a very low frequency in, population databases.
  - **Rationale:** The variant has an allele count of 2 in gnomAD v4.1.0 (Allele Frequency = 0.000001239), which is considered extremely rare and meets the criteria for this rule at a supporting level for recessive diseases.
- **PM3_Strong:** For recessive disorders, detected in *trans* with a pathogenic variant.
  - **Rationale:** The variant was confirmed to be in a *trans*-configuration with the pathogenic loss-of-function variant c.299dup.
- **PM6:** Assumed *de novo*, but without full maternity and paternity confirmed.
  - **Rationale:** The variant was absent in the available mother, sister, and daughter. The variant was detected only in blood. As the father was unavailable for testing, this criterion is applied as "Moderate" rather than "Very Strong".
- **PP3:** Multiple lines of computational evidence support a deleterious effect on the gene or gene product.
  - **Rationale:** The REVEL score for this variant is 0.77, which is above the commonly used threshold of 0.75 for predicting a damaging effect.
- **PP4:** The patient’s phenotype or family history is highly specific for a disease with a single genetic etiology.
  - **Rationale:** The clinical and biochemical presentation of ADP is highly specific and is exclusively associated with pathogenic variants in the *ALAD* gene.

**Final Classification:** The combination of 1 Strong (PM3), 2 Moderate (PM2, PM6), and 2 Supporting (PP3, PP4) criteria provides sufficient evidence to classify this variant as **Pathogenic**.

**Variant 2 (Germline)**

HGVS Nucleotide: c.299dup

HGVS Protein: p.(Ala101SerfsTer3)

Genomic Coordinates (hg38): chr9:113390895T>TG

Variant Type: Frameshift (duplication)

Zygosity in Proband (Blood & Fibroblasts): Heterozygous

Method of Detection: Sanger sequencing, Whole Exome Sequencing (WES), Whole Genome Sequencing (WGS).

**ACMG/AMP Classification: Pathogenic**

**Applied Criteria:** PVS1 + PM2_Moderate + PP4

**Justification of Applied ACMG/AMP Criteria:**

- **PVS1:** Null variant (frameshift) in a gene where loss-of-function (LoF) is a known mechanism of disease.
  - **Rationale:** ADP is a recessive disorder caused by LoF of the ALAD enzyme.
- **PM2_Moderate:** Variant is absent from population databases.
  - **Rationale:** The variant is novel and was not found in gnomAD or other control databases, meeting this criterion at a "Moderate" strength level.
- **PP4:** The patient’s phenotype is highly specific for ADP.
  - **Rationale:** As with the first variant, the patient's specific clinical and biochemical profile strongly points to variants in the *ALAD* gene.

**Final Classification:** The combination of 1 Very Strong (PVS1) and 1 Moderate (PM2) criteria is sufficient to classify this variant as **Pathogenic**.

**Configuration of Alleles:**

WES and long-read WGS analyses confirmed that the two variants are in a trans-configuration.
